# Medical Legislation

**Published:** 1901-03

**Authors:** 


### MEDICAL LEGISLATION.

### A BILL

TO BE ENTITLED.

An Act to repeal Title LXXXII, of the Revised Statutes of the State of Texas, and to pass in lieu thereof this act: To license physicians and surgeons and endeavor to regulate the practice of medicine, and to punish persons violating the provisions thereof in the State of Texas.

Be it enacted by the Legislature of the State of Texas:

SECTION 1. That Title LXXXII, Articles 3777, 3778, 3779, 3780, 3781, 3782, 3783, 3784, 3785, 3786, 3787, 3788, 3789, of the Revised Statutes of Texas, be and are hereby repealed, and the following be enacted:

SEC. 2. There shall be established three boards of medical examiners for the State of Texas, to be named and styled the Board of Medical Examiners for the State of Texas, The Board of Eclectic Medical Examiners for the State of Texas, and the Board of Homeopathic Medical Examiners for the State of Texas, said boards to consist of nine members each, whose term of office shall be for two years or until their successors have been appointed and qualified; provided, that no member shall be a professor or teacher in any medical school.

SEC. 3. The said boards shall each consist of men learned in medicine and surgery now in active practice, who shall have resided in the State of Texas, and shall have practiced medicine for not less than five years prior to their appointment, having complied with the laws relating to the practice of medicine already in force in the State of Texas, and shall be appointed by the Governor on the tenth day of May following his inauguration, from a list of names twice the number to be appointed, to be furnished him and recommended by the Texas State Medical Association, the Eclectic Medical Society of the State of Texas, the Homeopathic Medical Society of the State of Texas; provided, however, in case the Governor shall consider any of the persons so recommended unsuitable he may decline to appoint any such person or persons, and communicate the fact to the presiding officer of the society presenting the nomination, and such society shall, within sixty days thereafter, make other recommendations; and provided that no one shall be eligible to appointment or service on such medical board who is addicted to intoxication or the habitual use of morphine, cocaine or other such drugs. Vacancies occurring in such board shall be filled from the list already in the hands of the Governor for unexpired terms.

SEC. 4. The members of such board shall qualify by taking the usual oath of office before the county judge of the county in which they shall respectively reside. The officers of said boards shall be a president, vice-president, and secretary, who shall also act as treasurer. Six members shall constitute a quorum. Regular meetings of the boards shall be held at least twice a year at such times and places as the boards may from time to time determine. Due notice of said meeting shall be given by publication in such papers as may be selected by the boards. Special meetings may be held upon the call of the presidents and two members. The boards may prescribe rules, regulations and by-laws for their own proceedings and government, and for the examination by its members of applicants for the practice of medicine, surgery and midwifery.

SEC. 5. It shall be the duty of said boards at any of their meetings to examine all persons making application to them who shall desire to commence the practice of medicine, surgery, midwifery in this State, and who shall not by the provisions of this act be exempt from such examination, and when an applicant shall have passed a satisfactory examination a certificate signed by all members of the board shall be issued to said applicant entitling him or her to practice medicine, surgery, obstetrics, in the State of Texas, and shall have affixed to it the seal of the State of Texas.

SEC. 6. In case any applicant shall fail to pass a satisfactory examination he or she shall not be permitted to stand any further examination within one year thereafter, and in no event shall an applicant who stands rejected by one of said boards be examined or licensed by either of the other boards. If an applicant desires to practice a system not represented by any of the boards hereby established, he or she may elect for himself or herself the board before which he or she will appear for examination; provided, that no applicant shall be rejected because of his or her adherance to any particular school of medicine or system of practice, nor on account of his or her views as to the method of treatment, and cure of disease; and provided further, when in the opinion of the presidents of the boards any applicant has been unavoidably prevented from appearing before the board at their regular meeting, said president shall, upon notification, appoint a committee of three members to examine such applicant and if the examination be satisfactory, notify the secretary, who shall issue him or her a temporary certificate, which shall have the same force and effect as though granted by the full board until the applicant shall have the opportunity to appear before the board at its next regular meeting, when said temporary certificate shall become void. No applicant shall be admitted to examination who cannot submit satisfactory evidence that he or she is more than twenty-one years of age, and is of good moral character. Applications for license must be made in writing and presented to the president or secretary of the board before which the applicant desires to appear and must be accompanied with a fee of fifteen dollars; provided when the applicant desires to practice midwifery alone the fee shall be five dollars.

SEC. 7. The boards of examiners shall keep a record of their proceedings in a book kept for that purpose, showing name, age, place and duration of residence of each applicant, the time spent in medical study in or out of medical schools which have granted said applicant any degree or certificate of attendance upon lectures in medicine; said register shall show also whether said applicant was rejected or licensed and shall be prima facie evidence of all matter contained therein.

SEC. 8. From and after the passage of this amendment it shall be unlawful for any person to practice medicine, surgery or obstetrics in this State except: First, all those who were practicing medicine in Texas prior to January 1, 1885. Second, all those who began the practice of medicine in this State after the above date who have complied with the laws of this State regulating the practice of medicine in force prior to the passage of this act; provided that those who had diplomas recorded since January 1, 1891, shall present to the State boards of medical examiners herein provided for, satisfactory evidence that their diplomas were issued by bona fide medical colleges of reputable standing, receive a certificate from said boards, which shall be recorded as herein provided for, and provided that no fees shall be required for the issuing of such certificates. Third, all persons who shall hereafter receive certificates from the board of medical examiners of this State as above provided for, and who shall also in all other respects have complied with the provisions of this act. Fourth, and provided that all persons who may change their residence to the State of Texas, on filing a true copy of a license granted by the board of medical examiners of another State or Territory, certified by the affidavits of the president and secretary of said board with satisfactory proof of the genuineness of the same, and showing that the standard of requirements of the medical laws of said State or Territory and that adopted by said board of medical examiners are equal to that provided for in this act, and who, on the payment of the usual fee of fifteen dollars, may be registered and receive a license from the board of medical examiners of Texas to practice in this State. Fifth, and provided further, that all persons who desire to hereafter begin the practice of midwifery in this State and charge for their services shall make application to the medical examining boards, said applications to be accompanied with the fee of five dollars as herein provided, and when the said medical examining boards may admit to examination such applicants, and after passing a satisfactory examination in this special branch will be granted a license to practice midwifery in the State of Texas; provided, this should not apply to those who do not follow midwifery as a profession, and who do not advertise themselves as midwives, or hold themselves out to the public as practicing the profession of midwifery.

SEC. 9. Any person shall be regarded as practicing medicine or surgery within the meaning of this act who shall profess publicly to be a physician or surgeon and shall offer for practice as such or prescribe for those needing medical or surgical aid, and shall charge or receive therefor money or other compensation. This act shall be so construed as to include persons not pretending to be physicians who offer for sale publicly on the streets or other public places remedies not manufactured and compounded within this State, which they recommend for the cure of disease, but this act shall not apply to any commissioned officer or contract surgeon of the United States army, navy or marine hospital service in the performance of their duties as such, nor to any legally qualified and registered dentist under the laws of this State, nor to any lawfully qualified physician or surgeon residing in other States or territories, meeting registered physicians and surgeons of this State in consultation.

SEC. 10. The applicant shall be examined in the following branches: Anatomy, physiology, chemistry, materia medica, therapeutics, histology, pathology, practice of medicine, surgery, including diseases of the eye, ear, nose and throat; obstetrics, gynecology, hygiene and medical jurisprudence.

SEC. 11. The fund realized from the fees aforesaid shall be applied first to the payment of the necessary expenses of the board of examiners, each board receiving only such fees as are collected from applicants which appear before said board. In addition to expenses the secretary shall receive ten dollars per day while in actual attendance upon regular meetings. If any balance be left in the treasury of the boards it shall be divided among the members in attendance as compensation for loss of time.

SEC. 12. Before any person obtaining a license under the provisions of this act can lawfully practice medicine, surgery or midwifery in this State he or she shall cause the said license to be recorded by the clerk of the district court in a book to be kept for that purpose, which shall be properly indexed. The clerk shall receive from the applicant a fee of fifty cents for recording this instrument.

SEC. 13. Any person who shall practice medicine or surgery or midwifery in this State in violation of the provision of this act shall be fined not less than fifty nor more than five hundred dollars for each offense or by fine and imprisonment not exceeding six months and it shall not be lawful for him or her to recover by action, suit, motion, or warrant any compensation for services which may be claimed to have been rendered by him or her as such physician, surgeon, or midwife; provided, that the provisions of this act do not apply to persons treating disease who do not prescribe or give drugs or medicine.

SEC. 14. That Title LXXXII of the Revised Statutes of Texas, and Articles 3777, 3778, 3779, 3780, 3781, 3782, 3783, 3784, 3785, 3786, 3787, 3788, 3789, of said title be and the same are hereby repealed, and all laws or parts of laws in conflict with this act be and are hereby repealed.

SEC. 15. The fact that there is no law in force adequately providing for the license of physicians, surgeons and midwives creates an emergency and an imperative public necessity that the constitutional rule requiring bills to be read on three several days be suspended and the bill be placed on its final passage and it is so enacted.

